# SmileFinder: a resampling-based approach to evaluate signatures of selection from genome-wide sets of matching allele frequency data in two or more diploid populations

**DOI:** 10.1186/2047-217X-4-1

**Published:** 2015-01-14

**Authors:** Wilfried M Guiblet, Kai Zhao, Stephen J O’Brien, Steven E Massey, Alfred L Roca, Taras K Oleksyk

**Affiliations:** Biology Department, University of Puerto Rico at Mayagüez, Mayagüez, 00680 Puerto Rico; Department of Animal Sciences, University of Illinois at Urbana–Champaign, Urbana, 61801 Illinois USA; Theodosius Dobzhansky Center for Genome Bioinformatics, St. Petersburg University, St. Petersburg, 199034 Russia; Oceanographic Center, Nova Southeastern University, Ft. Lauderdale, 33004 Florida USA; Biology Department, University of Puerto Rico at Rio Piedras, Rio Piedras, 00931 Puerto Rico

**Keywords:** Genome, Selection, Resampling, Evolution, Population, Galaxy

## Abstract

**Background:**

Adaptive alleles may rise in frequency as a consequence of positive selection, creating a pattern of decreased variation in the neighboring loci, known as a selective sweep. When the region containing this pattern is compared to another population with no history of selection, a rise in variance of allele frequencies between populations is observed. One challenge presented by large genome-wide datasets is the ability to differentiate between patterns that are remnants of natural selection from those expected to arise at random and/or as a consequence of selectively neutral demographic forces acting in the population.

**Findings:**

*SmileFinder* is a simple program that looks for diversity and divergence patterns consistent with selection sweeps by evaluating allele frequencies in windows, including neighboring loci from two or more populations of a diploid species against the genome-wide neutral expectation. The program calculates the mean of heterozygosity and F_ST_ in a set of sliding windows of incrementally increasing sizes, and then builds a resampled distribution (the baseline) of random multi-locus sets matched to the sizes of sliding windows, using an unrestricted sampling. Percentiles of the values in the sliding windows are derived from the superimposed resampled distribution. The resampling can easily be scaled from 1 K to 100 M; the higher the number, the more precise the percentiles ascribed to the extreme observed values.

**Conclusions:**

The output from *SmileFinder* can be used to plot percentile values to look for population diversity and divergence patterns that may suggest past actions of positive selection along chromosome maps, and to compare lists of suspected candidate genes under random gene sets to test for the overrepresentation of these patterns among gene categories. Both applications of the algorithm have already been used in published studies. Here we present a publicly available, open source program that will serve as a useful tool for preliminary scans of selection using worldwide databases of human genetic variation, as well as population datasets for many non-human species, from which such data is rapidly emerging with the advent of new genotyping and sequencing technologies.

## Findings

### Rationale

With the advent of next generation sequencing and high-throughput genotyping technologies, it is now possible to evaluate patterns of frequency distributions of alleles along chromosomes, and look for signatures of selection in population data. In the simplest case, when a genetic variant is adaptive, it rises in frequency accompanied by nearby hitchhiking alleles, creating a pattern of decreased heterozygosity – this region is known as a selective sweep (reviewed in Hurst [1]). When allele frequencies from a set of loci inside the region affected by the sweep are compared to exactly the same region in a related population with no history of selection, a difference in allele frequencies between populations is observed. This change can be measured by an increased F_ST_[2] where small values close to 0.0 are interpreted as no difference between allele frequencies in the population (no genetic structure); while an F_ST_ of 1.0 is an indication of extreme population differentiation. An F_ST_ value can be calculated for a series of loci and averaged across the region, this is known as a multi-locus F_ST_. The fluctuating F_ST_ values (between large and small values) in sequentially sampled loci suggests a fixation of alternative alleles in a compared population, as haplotypes containing a variant targeted by selection may differ between them [3]. This fluctuation can be captured by the multi-locus F_ST_ variance (or S^2^F_ST_): the variance among the F_ST_ values for all loci (n = 5, 7, 9 …, k) contained in each sliding window. It has been previously argued that the S^2^F_ST_ is more useful for detecting signatures of selection, since the F_ST_ mean or median may decrease when high and low values for alternatively fixed alleles across a window are combined [3].

**Figure 1:**
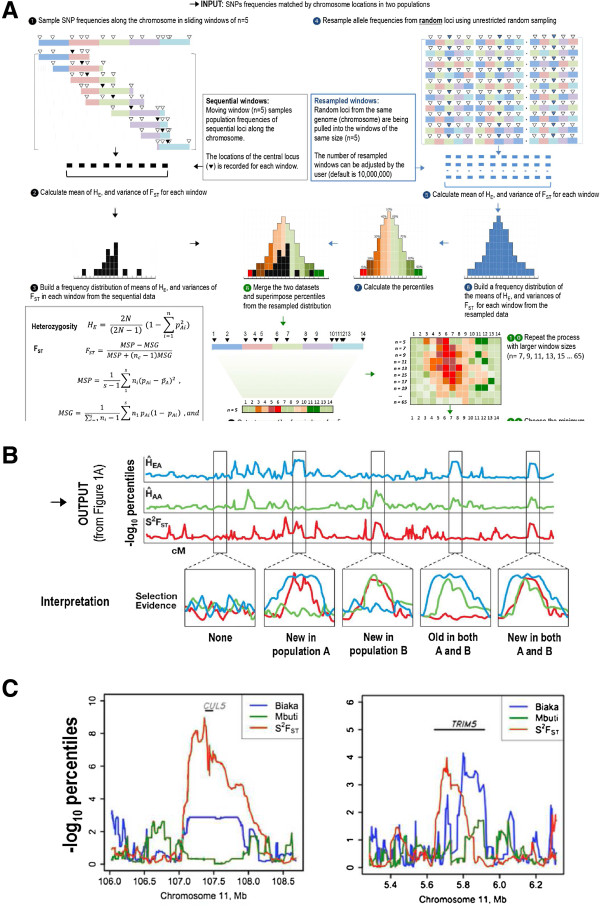
**Smilefinder program description, output and outcome examples. A**. Description of the *SmileFinder* algorithm. The program finds chromosomal regions with patterns of selection by comparing distributions of allele frequencies chromosome-wide in two or more populations, and infers the most extreme percentile values for each SNP from a resampled distribution representing a baseline approximating the neutral scenario. The program (1) takes an input of allele frequencies from two or more population and (2) samples along the chromosome sequential loci using a sliding window of n = 5. (3) At the same time, the program combines allele frequencies into sets from random loci using unrestricted random sampling (r = 10 K, 100 K, 1 M, 10 M or 100 M). (4) The algorithm then calculates mean and variance of Heterozygosity and F_ST_ in each window and the resampled set, and (5) builds a frequency distribution to (6) calculate the percentiles that are (7) superimposed onto the observed distribution. (8) The inferred percentiles are deposited into the output, then the process is repeated with incrementally larger window sizes (5, 7, 9, 11, 13, … , 65). (9) Percentiles are combined across all the different sized windows, and (10) the maximum value is chosen for the visual inspection of the data. **B**. The output can be plotted chromosome-wide to help find four patterns of putative regions for signatures of positive selection (modified from Oleksyk et al. [3]). Percentiles have been transformed for visualization: -log10 percentiles = log_10_ (1/percentile). **C**. The outcomes of a selection scan with *SmileFinder* algorithm indicating possible selection in two genes, *CUL5* and *TRIM5*, in Biaka populations from central Africa (modified from Zhao et al. [4]). The position of the genes on chromosome 11 are given in megabases (Mb).

While selection initially acts on the entire chromosome due to continuous crossover events, regions encompassing the sweeps become smaller with each generation of recombination. Because historic selection in the genome is localized, the frequencies of alleles drawn from the non-affected part of the genome can be assumed to reflect mostly neutral forces. A theoretical baseline distribution can be built from a very large number of random sets combining allele frequencies from multiple loci. Comparing values from sets of sequentially located loci to the matching sets from the resampled distribution can be used to superimpose the expected percentiles from the resampled distributions to real ones. In other words, we can assign expectation values to observed combinations using a distribution generated from the same data in a neutral scenario (Figure 1).

Here we present a simple tool that allows the identification of candidate selection regions in genome-wide allele frequency data by evaluating regional heterozygosity and frequency differences (F_ST_ variance) in sequential loci between two or more populations [3, 4]. The resampling approach can be applied to studies in any diploid species given matching single nucleotide polymorphism (SNP) allele frequency coverage in at least two populations. In a previously published study [3], this original strategy was designed and implemented to discover selective sweeps using two lightly genotyped (<200 K loci) human populations. A comparison to a dozen other methods showed that this method performs well by identifying simulated sweeps, and compared with nine other scans reported by other genome-wide scans available in the literature at the time [3]. In another study [4], the same algorithm was applied to demonstrate that genomes of primate hunting human populations in Africa are more likely to display selection signatures around the genes implicated in resistance in HIV and similar viruses. The current resampling scheme can incorporate other tests for evaluating selection signatures genome-wide [5].

## Functionality

### Program description

Here we present *SmileFinder*, a Python script that suggests and evaluates candidate regions containing patterns suggesting past actions of positive selection by comparing allele frequencies in datasets from two populations of diploid species. The program input is a list of locus names (such as rs#), location, values of heterozygosity for both populations, and the F_ST_ value between the two populations. The program calculates the mean heterozygosity and the S^2^F_ST_ in sliding windows and builds a resampled distribution (the baseline), using an unrestricted random sampling algorithm to randomize locations of each locus (Figure 1). Percentiles of observed value are derived from the randomized distribution. The observed distribution of loci in each window size is evaluated separately against the distribution of resampled sets containing the same number of random loci contained in the window, with the smallest window containing as few as five, and the largest containing as many as that specified by the user (the default is 65 loci, or 31 windows) centered on every SNP genotyped along the chromosome. The program output contains the most extreme percentiles (or extreme probabilities calculated using z-scores) for both heterozygosities and S^2^F_ST,_ assigned to the central coordinate in the sliding window. The current protocol is not intended to definitely prove the past selection in any given chromosomal region, but rather to only find candidate regions for historic selection genome-wide for downstream validation tests.

### Input

*SmileFinder* input is extremely simple. The data should be formatted in a plain text file with six required columns (see the example data in GigaDB [6]): locus name, chromosome, location, locus heterozygosity in population 1, locus heterozygosity in population 2, locus F_ST_. Loci with less than 10 genotypes in one population should be ignored. There are several worldwide databases of human genetic variation (The Human Genome Diversity Project [HGDP], 1000Genomes, etc.) from which allele frequency information and chromosome locations of the loci can be obtained [7, 8] A stand-alone code is provided to convert the format from the HGDP-type data file and to calculate heterozygosity and F_ST_ (count.py) [6]. To reduce the number of comparisons, populations should be compared in the context of the recent evolutionary history, accounting for the time of divergence [5]. Finally, the efforts of the Genome10K Consortium [9] and similar initiatives will inevitably lead to useful population datasets of genome-wide variation, while allele variation can already be filtered from Genotyping-By-Sequencing data [10].

### Workflow

The workflow of *SmileFinder* is presented as a flow chart in Figure 1A. The script looks for the chromosomal regions under selection by comparing distributions of heterozygosity and F_ST_ (chromosome-wide or genome-wide) for two or more populations to infer the most extreme percentile value for each SNP from a resampled distribution representing a baseline approximating the neutral scenario [3]. **(1)** The sliding windows are filled sequentially with the observed values (heterozygosity for each of the compared populations and the F_ST_) in two populations. **(2)** Random sets containing as many loci as the sliding window are generated using the unrestricted random sampling algorithm - by obtaining values from random locations along the chromosome. This sampling can be performed to generate distributions of 100 k, 1 M and 10 M values. **(3)** For each locus, mean heterozygosity and S^2^F_ST_ for each estimate is determined for values inside each window and each resampled set. **(4)** Real (sequential) multi-locus values from the windows are compared to the distribution of resampled random values (baseline), and **(5)** percentile is defined as the rank (equal or closest to the least extreme value) of the sequential value in the resampled set, divided by the number of values in the resampled set. **(6)** Different window sizes (default sizes are 5 to 65 by an increment of 2) centering on each locus along the chromosome are evaluated in the exact same manner, and **(7)** the most extreme percentile value is selected among the sliding window sizes. These values can be plotted chromosome-wide to help find candidate regions displaying signatures of selection (Figure 1B).

The percentiles adjusted for the baseline expected under the neutral scenario give a less biased estimate of multi-locus parameter deviations than the raw data, while the large number of resampling allows an estimation of the chance of observing each rare combination of sequential values without additional models or assumptions.

### Output and interpretation

The output contains the expected percentiles for the most extreme values for the mean heterozygosities and S^2^F_ST_ from each size sliding windows (31 windows by default) centered on the same locus for each locus genotyped with more than 10 samples in two populations (Figure 1C). A suggested interpretation of the observed data is outlined in Figure 1B (modified from Oleksyk et al. [3]). There are four possible outcomes. **(1)** No extreme values are observed. This outcome does not exclude presence of selection, the event may occur in the time that is not well captured by the methods based on allele frequencies, but other approaches can be used [5]. **(2)** A selection event potentially occurred in the ancestral population, 'old selection’, and produced a common selective sweep in the two derived populations. No elevated F_ST_ values are expected under this scenario. **(3)** If selection occurs only in one of the two derived populations, extreme deviations for multi-locus values of heterozygosity indicate a selective sweep. The average F_ST_ is expected to rise, as the difference between allele frequencies in the chromosomal region rise, while F_ST_ variance or S^2^F_ST_ captures alterations of high and low F_ST_ values in the selective sweep area. **(4)** Selection that occurred in both derived populations after the split, ‘new selection’, produces a common selective sweep in the two derived populations, but in this case, elevated F_ST_ values are expected, and high S^2^F_ST_ values are expected to indicate alterations between high and low F_ST_ in the region. F_ST_ alone is a poor estimator of selection signatures, and should be used in addition to other methods Oleksyk et al. [5]. In our case, F_ST_ variance is used to decide whether selection occurred before (old) the population separation or after (new). Figure 1C shows actual outcomes indicating possible selection in two genes, *CUL5* and *TRIM5*, in Biaka populations from central Africa (adopted from Zhao et al. [4]).

This algorithm may overlook many potential signatures of selection, particularly when the selected haplotype is smaller than the sliding window, and thus the resulting F_ST_ variance will be zero. Smaller windows (e.g. n = 5) may not be used if the average length of haplotypes is suspected to be smaller than the sliding window sizes. Since gene flow will reduce population differentiation and increase heterozygosity, it will probably not result in detection of false positives. Other genome effects have been previously modeled and explored [3], demonstrating loss of sensitivity with low selection and high recombination rates in the region.

### Further applications

The –log_10_ converted percentile (or probability) values can be plotted along the chromosome to look for patterns corresponding to one of the above scenarios (Figure 1B), or can be evaluated for outliers in a scatter plot (H_E_ vs. F_ST_, etc.). This strategy was implemented genome-wide to detect signatures of selection in two human populations, and then compared to nine other genome-wide scans for signatures of selection [3]. Although not widely available for every species, we recommend that physical locations may be converted into genetic distances to transform the percentile plots to account for varying recombination rates along the chromosomes [3]. When evaluating candidate regions using the *SmileFinder* algorithm, a list of candidate genes can be assigned percentile statistics in order to be compared with a larger set of randomly chosen genes. This approach has been recently used to show that HIV-1 may have shaped the genomes of some human populations in West Central Africa [4].

## Availability and requirements

Project name: SmileFinder

Project home page: https://github.com/wilfriedguiblet/smilefinder

Operating system(s): Platform independent

Programming language: Python

Other requirements: none

License: GPL v3

Any restrictions to use by non-academics: None

## Availability of supporting data

The SmileFinder script, a sample input dataset, and an accompanying instruction file are provided in GigaDB [6] and freely available to download from http://genomes.uprm.edu/smilefinder. The package is also fully integrated into the GigaGalaxy Server (http://galaxy.cbiit.cuhk.edu.hk/), and code freely available from GitHub (https://github.com/wilfriedguiblet/smilefinder). The dataset and software supporting the results of this article are available in the *GigaScience*, GigaDB repository [6].

## Abbreviations

SNP: Single nucleotide polymorphism.
